# Novel small-molecule compound YH7 inhibits the biofilm formation of *Staphylococcus aureus* in a *sarX*-dependent manner

**DOI:** 10.1128/msphere.00564-23

**Published:** 2024-01-03

**Authors:** Yanghua Xiao, Cailing Wan, Xiaocui Wu, Yanlei Xu, Yao Chen, Lulin Rao, Bingjie Wang, Li Shen, Weihua Han, Huilin Zhao, Junhong Shi, Jiao Zhang, Zengqiang Song, Fangyou Yu

**Affiliations:** 1 Department of Clinical Laboratory, Shanghai Pulmonary Hospital, School of Medicine, Tongji University, Shanghai, China; 2 School of Public Health, Nanchang University, Nanchang, China; 3 School of Pharmaceutical Sciences, Wenzhou Medical University, Wenzhou, China; NC State University, Raleigh, North Carolina, USA

**Keywords:** *Staphylococcus aureus*, antibiofilm, adhesion, PIA

## Abstract

The emergence of antibiotic-resistant and biofilm-producing *Staphylococcus aureus* isolates presents major challenges for treating staphylococcal infections. Biofilm inhibition is an important anti-virulence strategy. In this study, a novel maleimide-diselenide hybrid compound (YH7) was synthesized and demonstrated remarkable antimicrobial activity against methicillin-resistant *S. aureus* (MRSA) and methicillin-susceptible *S. aureus* (MSSA) in both planktonic cultures and biofilms. The minimum inhibitory concentration (MIC) of YH7 for *S. aureus* isolates was 16 µg/mL. Quantification of biofilms demonstrated that the sub-MIC (4 µg/mL) of YH7 significantly inhibits biofilm formation in both MSSA and MRSA. Confocal laser scanning microscopy analysis further confirmed the biofilm inhibitory potential of YH7. YH7 also significantly suppressed bacterial adherence to A549 cells. Moreover, YH7 treatment significantly inhibited *S. aureus* colonization in nasal tissue of mice. Preliminary mechanistic studies revealed that YH7 exerted potent biofilm-suppressing effects by inhibiting polysaccharide intercellular adhesin (PIA) synthesis, rather than suppressing bacterial autolysis. Real-time quantitative PCR data indicated that YH7 downregulated biofilm formation-related genes (*clfA*, *fnbA*, *icaA,* and *icaD*) and the global regulatory gene *sarX*, which promotes PIA synthesis. The *sarX*-dependent antibiofilm potential of YH7 was validated by constructing *S. aureus* NCTC8325 *sarX* knockout and complementation strains. Importantly, YH7 demonstrated a low potential to induce drug resistance in *S. aureus* and exhibited non-toxic to rabbit erythrocytes, A549, and BEAS-2B cells at antibacterial concentrations. *In vivo* toxicity assays conducted on *Galleria mellonella* further confirmed that YH7 is biocompatible. Overall, YH7 demonstrated potent antibiofilm activity supports its potential as an antimicrobial agent against *S. aureus* biofilm-related infections.

**IMPORTANCE:**

Biofilm-associated infections, characterized by antibiotic resistance and persistence, present a formidable challenge in healthcare. Traditional antibacterial agents prove inadequate against biofilms. In this study, the novel compound YH7 demonstrates potent antibiofilm properties by impeding the adhesion and the polysaccharide intercellular adhesin production of *Staphylococcus aureus*. Notably, its exceptional efficacy against both methicillin-resistant and methicillin-susceptible strains highlights its broad applicability. This study highlights the potential of YH7 as a novel therapeutic agent to address the pressing issue of biofilm-driven infections.

## INTRODUCTION

*Staphylococcus aureus* is a ubiquitous pathogen associated with bloodstream infections, endocarditis, pneumonia, keratitis, osteomyelitis, and skin soft tissue infections (1 – 3). The widespread prevalence of antibiotic resistance has restricted the clinical options available for treating *S. aureus* infection (4). Methicillin-resistant *S. aureus* (MRSA) infections are particularly challenging to eradicate because of their innate multidrug resistance (5). MRSA can adhere to medical devices and implant surfaces to form complex communities known as biofilms, making their treatment more challenging (6, 7).

Bacterial biofilms are intricate microbial aggregates comprising bacterial cells and a secreted extracellular matrix (ECM) (8). Compared with planktonic bacteria, biofilm-embedded bacteria exhibit a high degree of antibiotic resistance, can withstand various environmental stresses, and can evade the host immune system (8). Once bacteria form a biofilm on an implant surface, conventional antibiotic therapy often fails to eradicate these infections, which can persist over an extended period (9). The treatment of biofilm infections is further complicated by the high prevalence of clinical isolates of multidrug-resistant *S. aureus* (10). Vancomycin is the preferred treatment option for treating severe infections caused by MRSA; however, the bacteria have cleverly created an arsenal to resist vancomycin therapy (11). Thus, there is an urgent need to develop novel antibiofilm compounds that not only successfully prevent the formation of biofilms but also avoid bacterial resistance caused by the application of traditional antibiotics.

Se is an essential trace element involved in biological redox reactions and can inhibit microbial adhesion to surfaces (12). Selenium-containing compounds participate in biological redox reactions and can inhibit microbial attachment to solid surfaces (13, 14). Diphenyl diselenide compounds have been shown to possess anti-inflammatory, antioxidant, and antifungal activities (15, 16). Maleimide is an important structural core of the active molecular class of natural marine alkaloids, and extensive research on their derivatives in medicine indicates they have diverse bioactivities (17 – 19). Although both diphenyl diselenides and maleimide derivatives have demonstrated various medicinal properties, the antibiofilm activity and synergistic effects of mixtures of diselenide and maleimide compounds in *S. aureus* have not been reported. Therefore, we designed a novel diselenide maleimide hybrid, YH7, based on our previously developed metal-free and straightforward protocol for maleimide dissociation (20).

This study revealed the antibiofilm effect of YH7 and clarified its underlying mechanism. The *in vivo* and *in vitro* toxicity of YH7 was assessed. These findings underscore the therapeutic potential of YH7 in the treatment of *S. aureus* infections.

## MATERIALS AND METHODS

### Bacterial isolates, cells, and culture conditions

This study included a standard strain (NCTC 8325) and three clinical MRSA isolates (MR30, MR91, and MR273). These clinical MRSA isolates were used in our previous study and characterized using whole-genome sequencing (21). The sequencing results were deposited in GenBank under the accession numbers SAMN35068806, SAMN35068807, and SAMN35068808. We selected these isolates because of their strong biofilm-forming capabilities. Additionally, these MRSA isolates were recovered from clinical patients with different genetic backgrounds. MR30 was isolated from blood and belonged to sequence type 59 (ST59). MR91 was isolated from sputum and belonged to ST630. MR273 was isolated from pus and belonged to ST7. All isolates were cultured overnight in trypticase soy broth (TSB) at 37°C with shaking at 220 rpm. The A549 human lung cancer cell line used in this study was obtained from the American Type Culture Collection (Rockville, Maryland, USA). The human bronchial epithelial cell BEAS-2B was a gift from the Department of Anesthesiology, Shanghai Pulmonary Hospital, Tongji University School of Medicine (Shanghai, China). A549 lung epithelial cells and BEAS-2B cells were cultured in Dulbecco’s modified Eagle Medium (DMEM) supplemented with 10% fetal bovine serum under standard conditions of 37°C and 5% CO_2_.

### The synthesis of YH7

**Fig 1:**
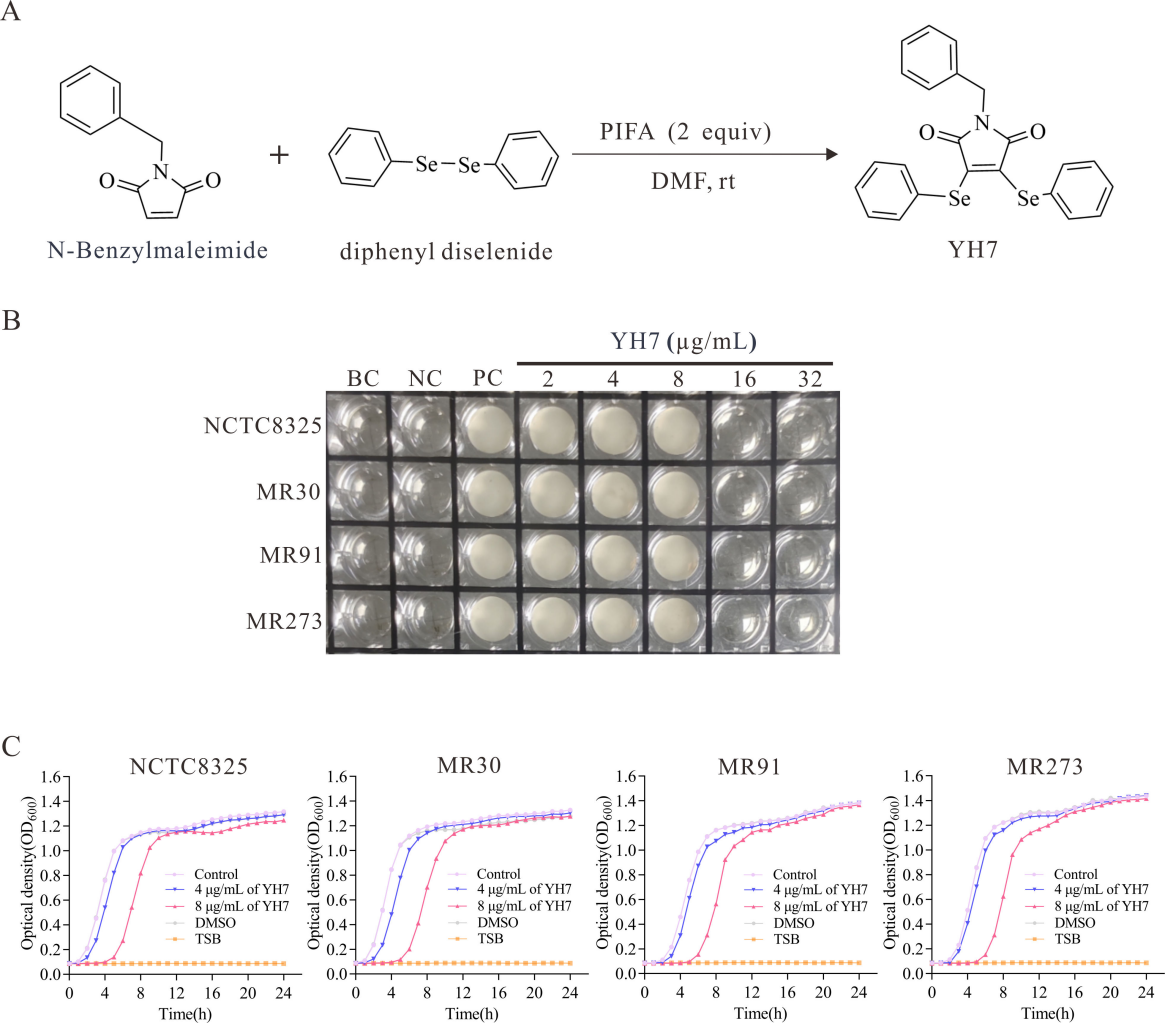
The synthetic process and antibacterial activity of YH7. (**A**) The synthetic process of YH7. PIFA, (bis(trifluoroacetoxy)iodo)benzene; DMF, dimethylformamide; RT, room temperature. (**B**) MIC result of YH7 against methicillin-susceptible *S. aureus* (NCTC8325) and MRSA (MR30, MR91, MR273) isolates using microdilution broth method. BC, blank control (CAMHB only); NC, negative control (CAMHB containing YH7 without *S. aureus*); PC, positive control (CAMHB containing *S. aureus* without YH7). (**C**) Growth curves of *S. aureus* isolates in the presence of different concentrations of YH7.

YH7 was synthesized by the Song laboratory at the School of Pharmacy, Wenzhou Medical University. The synthesis method of YH7 was performed as described previously (20) (Fig. 1A). N-benzyl maleimide (0.2 mmol), diphenyl diselenide (0.3 mmol), and bis(trifluoroacetoxy)iodo)benzene (172.0 mg, 0.4 mmol) were mixed in a tube containing 2 mL of dimethylformamide (0.1M). The tubes were incubated at room temperature for 1 h. After completion of the reaction, the resulting mixture was purified via chromatography with elution using a 5% EtOAc solution in petroleum ether, resulting in a yellow solid with a yield of 71% (70.6 mg). Dimethyl sulfoxide (DMSO) was used to dissolve the YH7. Further characterization of YH7 was conducted through nuclear magnetic resonance spectroscopy and high-resolution mass spectrometry. The results obtained were as follows: ^1^H NMR (400 MHz, CDCl_3_) δ 7.46 (d, *J* = 7.5 Hz, 4H), 7.37–7.26 (m, 11H), 4.62 (s, 2H) ppm; ^13^C{^1^H} NMR (101 MHz, CDCl_3_) δ 167.1, 138.4, 136.1, 134.6, 129.3, 128.8, 128.6, 128.6, 127.8, 126.1, 42.4 ppm; ^77^Se NMR (115 MHz, CDCl_3_) δ 355.58 ppm. HRMS (ESI): m/z calculated for [M + H] ^+^ C_23_H_18_NO_2_Se_2_: 499.9668; observed: 499.9664.

### Determination of minimum inhibitory concentration (MIC)

The microdilution broth method was used to determine the MIC of YH7. To obtain a 0.5 McFarland turbidity standard bacterial suspension, colonies were resuspended in saline and subsequently diluted 1:100 with cation-adjusted Mueller-Hinton broth (CAMHB). Using a 96-well plate, 100 µL of the bacterial suspension and 100 µL of CAMHB were added, incorporating various YH7 concentrations (ranging from 1 µg/mL to 256 µg/mL). DMSO was used as a control to account for any potential solvent effects. The plate was incubated under static conditions at 37°C for 24 h. The experiments were repeated three times to ensure accuracy.

### Growth assay

To prepare 0.5 McFarland standard bacterial suspensions, we harvested bacterial cells in the logarithmic growth phase. The bacterial suspensions were diluted 1:100 with TSB medium, and YH7 was added to the medium to obtain final concentrations of 4 µg/mL and 8 µg/mL. To account for the potential impact of the solvent, an equal volume of DMSO (8 µg/mL) was used as the control. Next, 200 µL of the mixture was transferred to each well of a sterile Bioscreen honeycomb plate. An automated microbial growth curve analyzer (Bioscreen, Finland) was used to continuously monitor the OD_600_ at 37°C for 24 h. The measurements were shown as averages across three replicate holes. The experiment was independently conducted thrice, yielding similar results.

### Resistance inducing assay

To evaluate the potential for *S. aureus* to develop resistance to YH7, a serial passaging method was employed, with MIC determination as above described (22). Briefly, *S. aureus* strains NCTC8325 and ATCC29213 were diluted 1:200 in CAMHB medium, and YH7 was added to achieve sub-MIC (0.5 × MIC). Bacterial growth in CAMHB medium without antimicrobial agents served as the control. Cultures were incubated at 37°C with agitation at 220 rpm for 16–24 h. After each passage, the MIC was re-determined. If an increase in MIC was observed, a new sub-MIC concentration of YH7 (0.5 × new MIC) was established for the next passage. This process was repeated for 20 passage to assess the potential for resistance over repeated exposure. Ciprofloxacin and rifampicin were employed as positive controls. Changes in MIC values after repeated passages were used to evaluate resistance development.

### Hemolysis assay

The hemolysis assay was conducted following a previously reported procedure, with minor modifications (23). One hundred microliters of YH7 at different concentrations in PBS (Phosphate-Buffered Saline) was mixed with 900 µL PBS containing 5% defibrinated rabbit erythrocytes (Nanjing Maojie Microbial Technology Co., Ltd., Nanjing, China), resulting in a final concentration of 4, 8, or 16 µg/mL, respectively. To establish a positive control, 1% Triton X-100 was used, whereas sterile PBS served as the negative control. Following incubation at 37°C for 1 h, erythrocytes were separated from the supernatant by centrifugation at 1,000 *g* for 5 min. Subsequently, the absorbance at 600 nm was recorded, and the hemolysis ratio was calculated using the following formula: hemolysis (%) = (A_sample_ − A_PBS_) / (A_Triton_ − A_PBS_) × 100%. The experiments were repeated thrice.

### Cytotoxicity assay

Cell viability was assessed using the cell counting kit 8 (KGA317, KeyGEN, Nanjing, China) method. Cytotoxicity assay was done as described before with minor modifications (24). Briefly, A549 and BEAS-2B cells were seeded at a density of 10^3^ cells/well in DMEM in a 96-well plate. After 24 h of incubation in a cell incubator, various concentrations of YH7 (0.25–128 µg/mL) were added to the cells. Wells that contain 0.3% DMSO and did not contain YH7 were used as the control. After incubation for 24 h, wells were carefully washed twice with sterile PBS. Next, 100 µL DMEM was added to each well, and 10 µL of CCK8 reagent was introduced in a dark environment. After 2 h of incubation at 37°C, OD_450_ was recorded using a microplate reader. The results were calculated as follows: Cell viability (%) = (OD_450_ sample value − OD_450_ blank hole) / (OD_450_ value of untreated control − OD_450_ blank hole) × 100%.

### Evaluation of YH7 toxicity to *Galleria mellonella* larvae

*G. mellonella* larvae have proven to be a suitable model for assessing the *in vivo* toxicity of novel antimicrobial agents (25). *G. mellonella* larvae (about 250 mg per larvae) were randomly divided into four groups, 15 larvae per group. Compound YH7 was diluted with sterile 0.9% (wt/vol) NaCl to obtain concentrations of 4, 8, and 16 µg/mL. Each larva was injected with 10 µL of varying YH7 concentrations (4, 8, 16 µg/mL), corresponding to 0.16, 0.32, and 0.64 mg/kg doses. The larvae received injections of 10 µL YH7 at different concentrations, while the control group was injected with 10 µL sterile 0.9% NaCl. Following inoculation, the larvae were carefully transferred to sterile petri dishes and incubated in a dark environment at 37°C. The survival rate of the larvae was monitored at regular intervals of 12 h until 5 d after injection.

### Biofilm semi-quantitative assay

*S. aureus* cells from an overnight culture in TSB at 37°C were diluted 200 times in TSBG (TSB with 0.5% glucose) supplemented with varying concentrations of YH7 (0, 2, 4, and 8 µg/mL). Then, 200 µL of the diluted culture was transferred to the individual wells of a 96-well polystyrene microtiter plate. Following incubation for 24 h at 37°C, the wells were washed thrice with PBS to remove planktonic bacteria. The biofilms were immobilized by adding 100 µL of 100% methanol per well for 10 min, followed by staining with 100 µL of crystal violet (Beyotime) per well for 10 min. The excess dye was then rinsed off with tap water, and the plate was left to air-dry before capturing images for observation. Crystal violet was subsequently solubilized in 200 µL of 30% acetic acid per well, and the OD_600_ was measured using a microplate reader. All experiments were performed in triplicate. The biofilm formation image for the wild-type NCTC8325 data in Fig. 5A is the same as the image in Fig. 3A, as both were obtained from the same experiment.

### Confocal laser scanning microscopy (CLSM)

*S. aureus* isolates were cultured in confocal dishes (Biosharp, BS-15-GJM, China) with TSBG containing varying concentrations of YH7. Confocal dishes without YH7 were used as the controls. After culturing for 24 h at 37°C, dishes were washed twice with PBS. Subsequently, the biofilms were stained with SYTO9 and propidium iodide (PI) reagents, according to the manufacturer’s instructions (L7012, Thermo Fisher, USA). The dishes were incubated in the dark for 30 min. Subsequently, the biofilm-stained samples were observed using CLSM (Leica, Wetzlar, Germany).

### Cell adhesion assay

Cell culture assays were conducted as previously described, with slight modifications (26). Approximately 5 × 10^5^ cells were seeded in individual wells of 12-well plates. After the cell monolayer had formed, bacteria (MOI (the multiplicity of infection) = 10 bacteria/cell) in DMEM, with or without 4 µg/mL YH7, were used to replace the culture medium and then incubated at 37°C for 1, 2, and 3 h. After each incubation step, the A549 cells were carefully washed with sterile PBS. Subsequently, the cells were trypsinized and lysed using 0.05% Triton X-100. Bacterial counts were determined by plating serial dilutions of the cell lysates on tryptic soy agar plates after overnight incubation. The percentage of adhesion inhibition was calculated by comparing the average number of adhered bacteria per well to that of the untreated wells. All experiments were performed in triplicate.

### Murine nasal tissue colonization model

The nasal tissue colonization experiment was performed as described previously with slight modifications (27). Briefly, 5-week-old BALB/c mice (female, approximately 20 g) were randomly divided into two groups of seven mice each. Mice in each group were injected intravenously with either 200 µL of YH7 (total dose of 0.8 mg/kg) or PBS (control) 1 h prior to bacterial inoculation. Then, 30 µL of suspension containing 1.5 × 10^7^ CFU (Colony-forming unit) of *S. aureus* NCTC8325 was slowly dropped into the nasal cavities of the mice. After 48 h, the mice were euthanized. Their nasal tissues were excised after surface decontamination with 70% ethanol. The tissues were homogenized and dilutions were plated on blood agar to enumerate CFUs after overnight incubation. Bacterial burden in the nasal tissues was assessed by comparing CFU counts between the treatment and control groups.

### Quantification of polysaccharide intercellular adhesin (PIA) by immunoblot analysis

PIA quantification was performed as previously described with slight adjustments (28). The bacteria were diluted 1:100 in 0.5% TSBG with or without 4 µg/mL YH7 and incubated in a six-well plate at 37°C for 24 h. Next, the wells were washed three times with PBS to remove planktonic bacteria. Subsequently, 500 µL of EDTA (0.5 M) was added, and the mixture was kept on ice for 1 h. Surface-attached bacteria (biofilms) were collected by scraping. Equal numbers of bacterial cells were obtained by centrifugation. Bacteria were boiled at 100°C for 10 min and centrifuged at 13,000 rpm for 10 min. For each sample, 40 µL of the supernatant was mixed with 20 µL of proteinase K (20 mg/mL). Next, 10 µL of the sample was spotted on the negative control membrane. The membrane was dried and blocked with 5% BSA (Bovine serum albumin, Biosharp, Beijing, China) overnight at 4°C. Following washing with PBST (Phosphate-Buffered Saline with 0.1% Tween-20), the membranes were incubated at 37°C for 1 h with HRP (Horseradish peroxidase)-conjugated wheat germ agglutinin. Following another three washes (10 min each) with PBST, the membranes were developed using enhanced chemiluminescence (ECL Plus; Beyotime). The PIA image for the wild-type NCTC8325 data in Fig. 5A is the same as the image in Fig. 3A, as both were obtained from the same experiment.

### Quantitative analysis of extracellular DNA (eDNA)

Isolation and quantification of eDNA were performed as previously described (29). *S. aureus* isolates were cultured in six-well plates as previously described. Following biofilm formation and subsequent treatment, the biofilms were carefully resuspended in 500 µL of EDTA (0.5 M) and placed on ice for 1 h. The biofilms were resuspended in freshly prepared TES buffer (50 mM Tris-HCl, pH 8.0; 10 mM ETDA, and 500 mM NaCl). The samples were centrifugation at 16,000 rpm for 5 min, and the resulting supernatants were transferred to new tubes. Equal volumes of phenol-chloroform-isoamyl alcohol (25:24:1) and chloroform-isoamyl alcohol (24:1) were added to the supernatant. After thorough mixing, the tubes were stored at −20°C overnight, with the addition of 10% 3M Na-acetate in ethanol. Subsequently, eDNA was collected by centrifugation at 16,000 rpm for 20 min, washed with 75% ethanol, and dissolved in TE buffer. Quantification of eDNA was performed using a Nanodrop 2000 spectrophotometer (Thermo Fisher Scientific). To determine relative eDNA secretion, the total eDNA (ng) was divided by the OD_600_ of the biofilm.

### Determination of Triton X-100-induced autolysis

The autolysis test was conducted following a previously established method with minor adjustments made to enhance precision (30). Overnight cultures were introduced into 50 mL of TSB containing 1 M NaCl at a ratio of 1:200. Once the culture reached an OD_600_ of 0.2–0.3, one aliquot was treated with 4 µg/mL YH7. The cultures were then agitated until they reached an OD_600_ of 0.6, after which, they were centrifuged for 20 min at 4°C at 4,000 × *g*. The resulting bacterial pellet was gently washed with cold deionized water. The bacteria were resuspended in lysis buffer (pH7.2) containing 0.05% Triton X-100 and 50 mM Tris-Cl. The OD_600_ was carefully adjusted to 1.0, and this value was recorded at 30-min intervals for a duration of up to 3 h, with continuous shaking at 220 rpm. To ensure accuracy, all assays were performed in triplicate.

### Real-time quantitative PCR (RT-qPCR)

Extracting RNA from exponentially growing cells is essential for profiling the dynamic alterations in biofilm-related genes during the initial phases of *S. aureus* biofilm formation (8). Thus, *S. aureus* isolates were cultured in TSB at 37°C for 9 h under two conditions: with or without 4 µg/mL YH7. RNA extraction was performed using a Total RNA Purification Kit (Sangon Biotech). The PrimeScript RT Reagent Kit with gDNA Eraser (Takara) was used for reverse transcription. Quantitative PCR analysis was conducted using the QuantStudio 5 Real-Time PCR System with TB Green Premix (Takara). The RNA expression changes of the target genes were calculated using the formula 2−ΔΔ^Ct^ (31). The primer pairs used are listed in Table 1. Each reaction was performed in triplicate.

**TABLE 1:** Primer pairs used in this study

Primer	Sequence (5´–3´)
*gyrB*-RT-F	ACATTACAGCAGCGTATTAG
*gyrB*-RT-R	CTCATAGTGATAGGAGTCTTCT
*clfA*-RT-F	GCTTCAGTGCTTGTAGGTA
*clfA*-RT-R	GCTATCAGATTGCGTAACAC
*fnbA*-RT-F	TTCCTTAACTACCTCTTCT
*fnbA*-RT-R	CAATCATATAACGCAACAG
*icaA*-RT-F	GTTGGTATCCGACAGTATA
*icaA*-RT-R	CACCTTTCTTACGTTTTAATG
*icaD*-RT-F	ATGGTCAAGCCCAGACAGAG
*icaD*-RT-R	AGTATTTTCAATGTTTAAAGCA
*icaR*-RT-F	GGATGCTTTCAAATACCAACT
*icaR*-RT-R	TTATCTAATACGCCTGAGGAAT
*psmα*-RT-F	GTATCATCGCTGGCATCA
*psmα*-RT-R	AAGACCTCCTTTGTTTGTTATG
*psmβ*-RT-F	CGCAATTAAAGATACCGTAACTGCAGC
*psmβ*-RT-R	ACCTAATAAACCTACGCC
*sarX*-RT-F	AACATTGCTTGGCTTCTAT
*sarX*-RT-R	AATCTAGCTCATCCATTGC
*sarX*-Up-F	GGGGACAAGTTTGTACAAAAAAGCAGGCTAGCGACTTAAATTCGATTCGTTA
*sarX*-Up-R	CTATGCTTTCGACACTCAATTTCAAGTCTTTCTCATTTGTTTTTAATACG
*sarX*-Down-F	CGTATTAAAAACAAATGAGAAAGACTTGAAATTGAGTGTCGAAAGCATAG
*sarX*-Down-R	GGGGACCACTTTGTACAAGAAAGCTGGGTTCCATTGTTCTGCTGATT
*sarX*-C-F	CGGAATTCCGACACCTTGATATGTATTGCA
*sarX*-C-R	CGGGATCCCGCATTATTGAACTACGATTTC

### Construction of *sarX* deletion mutants and complementation mutants

In summary, DNA fragments upstream and downstream of *sarX* were amplified from NCTC8325 chromosomal DNA. These PCR products were then fused using overlap extension PCR and joined to the pKOR1 vector through a BP reaction (Integrating linear DNA fragments into circular donor vectors in the Gateway Cloning System), leading to the creation of recombinant plasmids known as pKOR1-Δ*sarX*. Subsequently, these plasmids were transferred into *Escherichia coli* DH5α and DC10B cells and electroporation was performed on NCTC8325. To create a complementary mutant strain with a *sarX* deletion, the full-length *sarX* gene, along with its native promoter region, was amplified by PCR and ligated to the pRB473 vector using the T4 enzyme. The resulting complement plasmid, designated pRB473-*sarX*, was electroporated into a *sarX* deletion mutant. The primers used for these procedures are listed in Table 1.

### Statistical analysis

Data analysis was performed using GraphPad Prism v8.0 (GraphPad Software). The results are expressed as the mean ± standard deviation. The significance of differences was determined using an unpaired *t*-test. Statistical significance was considered at *P* < 0.05, and the results were denoted as follows: *P < 0.05, **P < 0.01, ***P < 0.001, ****P < 0.001, and ns, *P* > 0.05.

## RESULTS

### Influence of sub-MICs of YH7 on the growth of *S. aureus* isolates

The synthetic scheme for YH7 is shown in Fig. 1A. Compound YH7 displayed antibacterial activity against various *S. aureus* isolates, including NCTC8325, MR30, MR91, and MR273, with an MIC of 16 µg/mL (Fig. 1B). Two sub-MICs of YH7 (4 µg/mL and 8 µg/mL) were selected, and their inhibitory activity against staphylococcal isolates was evaluated by growth curve analysis. Growth curve analysis showed that 8 µg/mL of YH7 significantly inhibited bacterial growth (Fig. 1C). At a concentration of 4 µg/mL, YH7 had no significant effect on the growth of NCTC8325, MR91, and MR273. Only the MR30 isolate in the logarithmic growth phase was slightly inhibited by 4 µg/mL YH7 but did not affect the late logarithmic phase.

### YH7 does not induce drug resistance in *S. aureus* and demonstrates non-toxic to rabbit erythrocytes, human cells, and *G. mellonella* larvae at antibacterial concentrations

**Fig 2:**
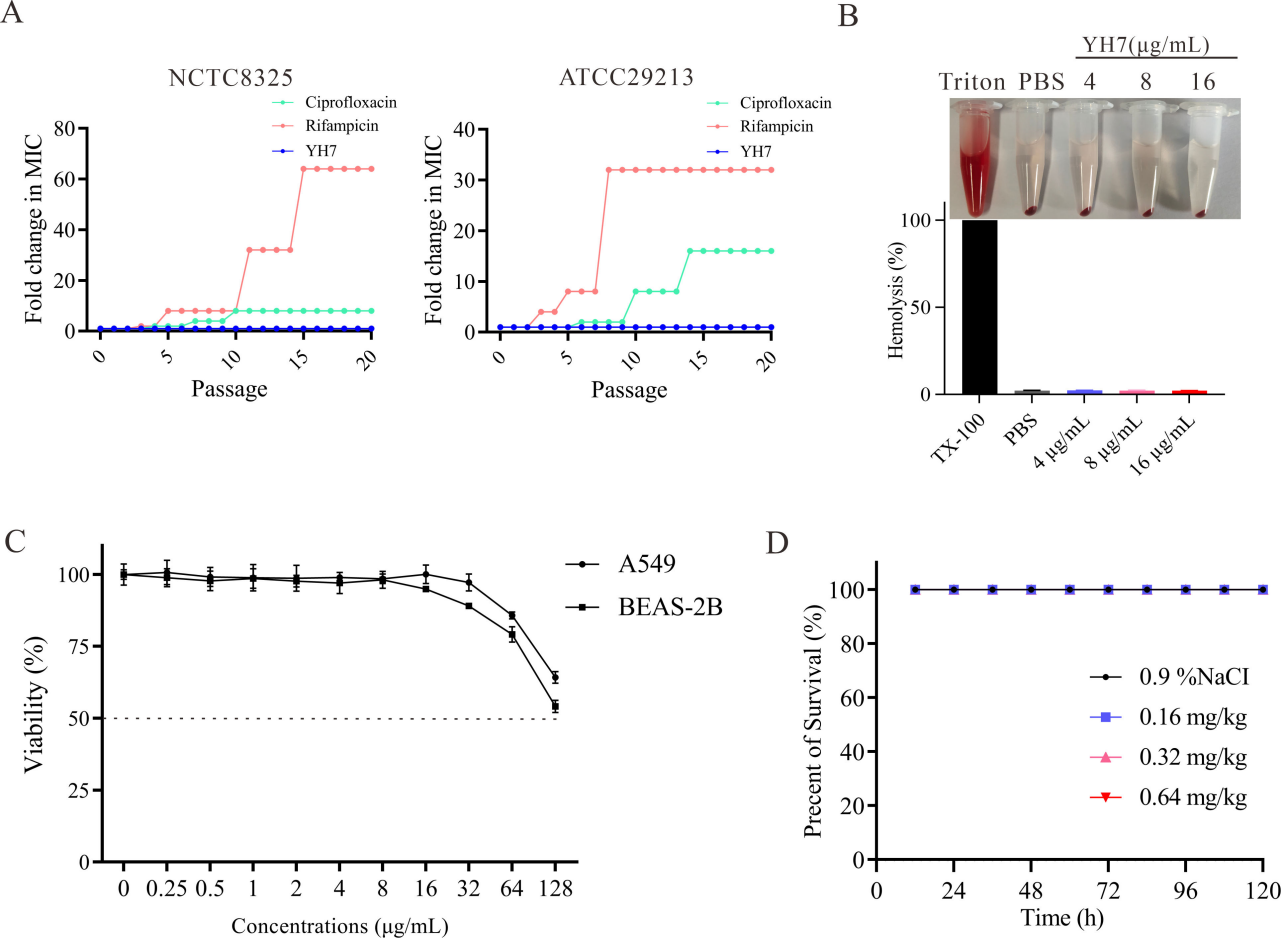
*In vitro* and *in vivo* toxicity of YH7. (**A**) A continuous resistance passage of sub-MIC of YH7, ciprofloxacin and rifampicin against *S. aureus* NCTC8325 and ATCC29213. (**B**) Hemolytic activity of YH7 on rabbit erythrocytes. (**C**) Cytotoxicity of various concentrations of YH7 on human A549 and BEAS-2B cells. (**D**) *In vivo* toxicity of YH7 on *G. mellonell*a larvae (*n* = 15 per group).

To assess the potential of YH7 to induce drug resistance in *S. aureus*, we measured MIC values at passages 0–20 during continuous subculturing with YH7 at sub-MIC. As shown in Fig. 2A, while the control antibiotics, ciprofloxacin and rifampicin, exhibited 8-fold to 64-fold increases in MIC against *S. aureus* strains NCTC8325 and ATCC29213 after 20 passages, the MIC of YH7 remained consistently unchanged throughout the experiment. These results suggest YH7 has a lower propensity to induce drug resistance in *S. aureus* compared to rifampicin and ciprofloxacin.

To confirm the biosafety of YH7, hemolysis, cytotoxicity, and *G. mellonella* assays were performed. After YH7 at different concentrations was incubated with rabbit erythrocytes, the level of hemoglobin detected in the supernatant was comparable to the baseline values obtained in the PBS control (Fig. 2B). This indicates that compound YH7 did not cause hemolysis of rabbit erythrocytes, even at its active antibacterial concentration. Cytotoxicity against human lung cell lines A549 (alveolar epithelial cells) and BEAS-2B (bronchial epithelial cells) was evaluated using the CCK-8 assay after 24 h of YH7 treatment. As presented in Fig. 2C, YH7 exhibited low cytotoxicity against both A549 and BEAS-2B cells, with IC50 (Half-maximal inhibitory concentration) values greater than 128 µg/mL. Additionally, we injected *G. mellonella* larvae with YH7 (0.16, 0.32, and 0.64 mg/kg) and monitored their survival status for 120 h. Consistent with the *in vitro* result, YH7 did not cause acute toxicity (Fig. 2D). These experimental data suggest that YH7 has good biocompatibility and considerable potential for clinical applications.

### YH7 inhibits *S. aureus* biofilm formation

**Fig 3:**
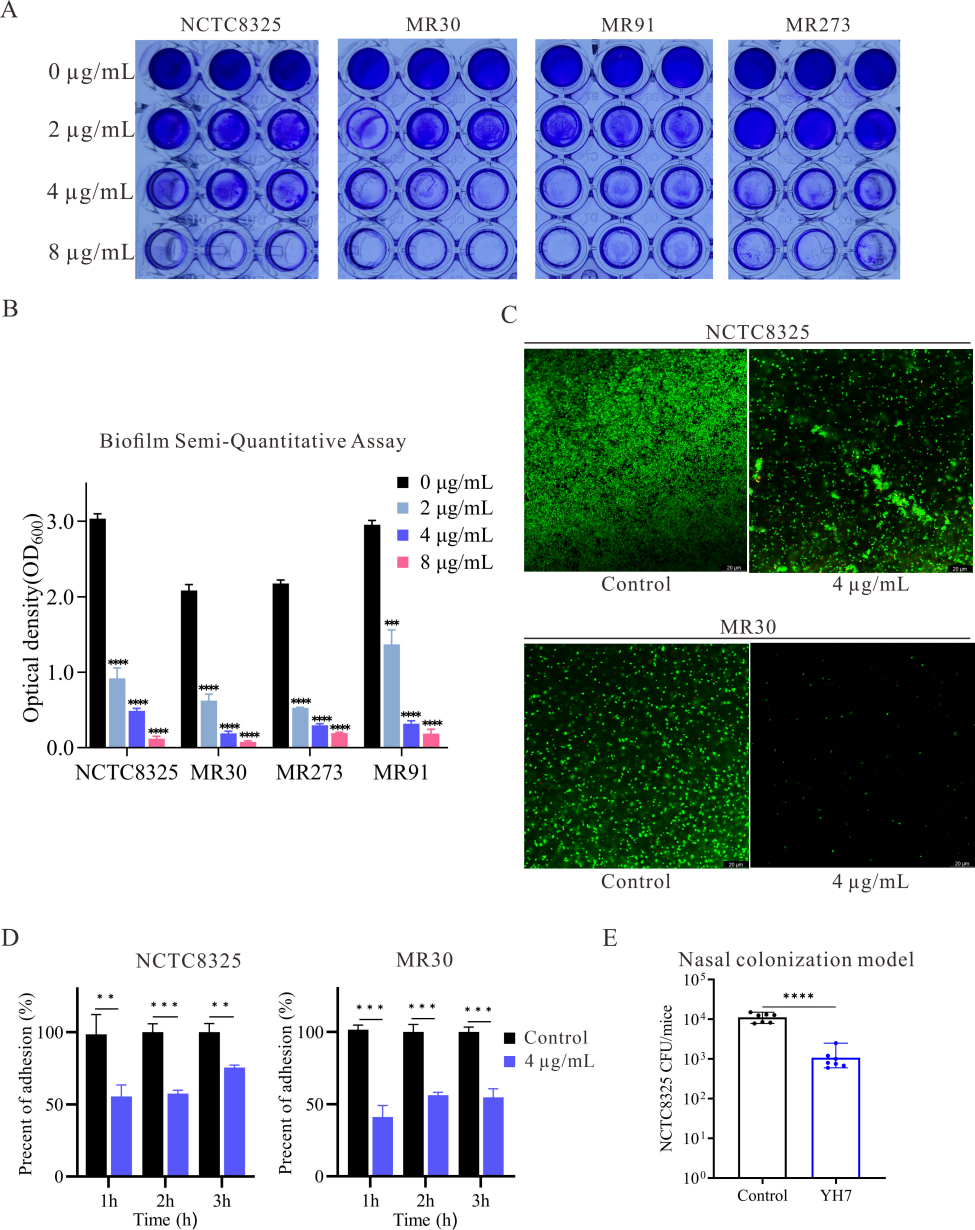
Antibiofilm and anti-adhesion performance of YH7. (**A**) Representative photographs of crystal violet-stained colonies are shown. (**B**) The optical density values at 600 nm were measured after the biofilms were dissolved with acetic acid. (**C**) CLSM images of biofilm in the presence of 4 µg/mL of YH7 for 24 h. (**D**) Inhibition of *S. aureus* adhesion to A549 cells in the presence of 4 µg/mL of YH7. The data are represented as the mean + SD of three replicates in a representative experiment chosen from three independent experiments. (**E**) Effect of YH7 (total 0.8 mg/kg) in decreasing *S. aureus* murine nasal colonization (*n* = 7/group). Data were collected from two independent experiments and the representative results were shown.

Previous studies have reported that selenium-containing compounds can inhibit the attachment of microorganisms to solid surfaces (13, 14). Thus, we hypothesized that YH7 affects biofilm formation. For subsequent experiments, we selected a concentration of 4 µg/mL YH7. The effect of YH7 on biofilm formation by *S. aureus* was quantified via crystal violet staining, which revealed that 4 µg/mL of YH7 significantly inhibited biofilm formation by methicillin-susceptible *S. aureus* (MSSA) and MRSA compared with untreated isolates (Fig. 3A). In the presence of YH7, measured OD_600_ corresponding to biofilm mass were significantly reduced. Specifically, OD_600_ decreased from 3.04 ± 0.06 to 0.49 ± 0.03 for NCTC8325, 2.09 ± 0.08 to 0.19 ± 0.03 for MR30, 2.18 ± 0.05 to 0.30 ± 0.02 for MR91, and 2.95 ± 0.06 to 0.32 ± 0.4 for MR273 (Fig. 3B). These differences were statistically significant (*P* < 0.001). To visualize the antibiofilm effect of YH7, we selected an MSSA strain (NCTC8325) and an MRSA strain (MR30) for CLSM analysis. To distinguish between live and dead cells in the biofilms, we used a fluorescent dye staining technique, employing SYTO9 dye to stain live bacteria green, while PI dye penetrated the membranes of dead cells, resulting in red coloration. As shown in Fig. 3C, CLSM revealed the limited presence of live cells and a few dead cells attached to the culture surfaces in the treated group. These results showed that YH7 effectively inhibits biofilm formation across both methicillin-susceptible and methicillin-resistant *S. aureus* strains at sub-MIC.

### YH7 decreased the adhesion force of *S. aureus* to A549 cells

*S. aureus* has emerged as a leading pathogen in nosocomial pneumonia (32). The A549 cell line is a widely used model for evaluating pulmonary diseases. Therefore, we investigated the inhibitory effect of YH7 on bacterial adhesion to A549 cells. The inhibitory effects of YH7 (4 µg/mL) on the adhesion of NCTC8325 and MR30 to A549 cells were tested at different times. As shown in Fig. 3D, 4 μg/mL of YH7 effectively reduced the adhesion of NCTC8325 to A549 cells after 1, 2, and 3 h compared to untreated cells (100%, 100%, and 100% vs 55.38%, 57.50%, and 75.49%, respectively). Similar results were observed for the effect of YH7 on MR30; 4 µg/mL YH7 significantly reduced MR30 adhesion to A549 cells over time compared to untreated cells (100%, 100%, and 100% vs 41.07%, 56.18%, and 57.72%, respectively).

### YH7 treatment reduces *S. aureus* colonization in the murine nasal tissue model

Bacterial adhesion to host tissues and biomaterial surfaces is the initial critical step in biofilm formation (33). High-level nasal carriage of *S. aureus* was a major risk factor for subsequent infection (34). Preventing *S. aureus* colonization and biofilm formation early could help reduce infection risks, particularly in high-risk populations like hospitalized patients who undergo cardiothoracic surgery (35). Given YH7 exhibited potent anti-biofilm activity *in vitro*, and the propensity for *S. aureus* to colonize and form biofilms in the human and murine nasal cavities (36), we employed the murine nasal colonization model to evaluate the efficacy of YH7 *in vivo*. Mice received an intravenous injection of either 200 µL YH7 (0.8 mg/kg total dose) or PBS (control) 1 h prior to intranasal inoculation with 30 µL suspension containing 1.5 × 10^7^ CFU *S*. *aureus* NCTC8325. CFU counts in nasal tissues 48 h post-inoculation revealed a statistically significant reduction (*P*＜ 0.001) in bacterial burden among YH7-treated mice compared to controls (Fig. 3E). These findings demonstrate YH7 significantly inhibits *S. aureus* colonization in the nasal cavities of murine model.

### YH7 inhibits *S. aureus* biofilm formation by reducing PIA production but not by eDNA release

**Fig 4:**
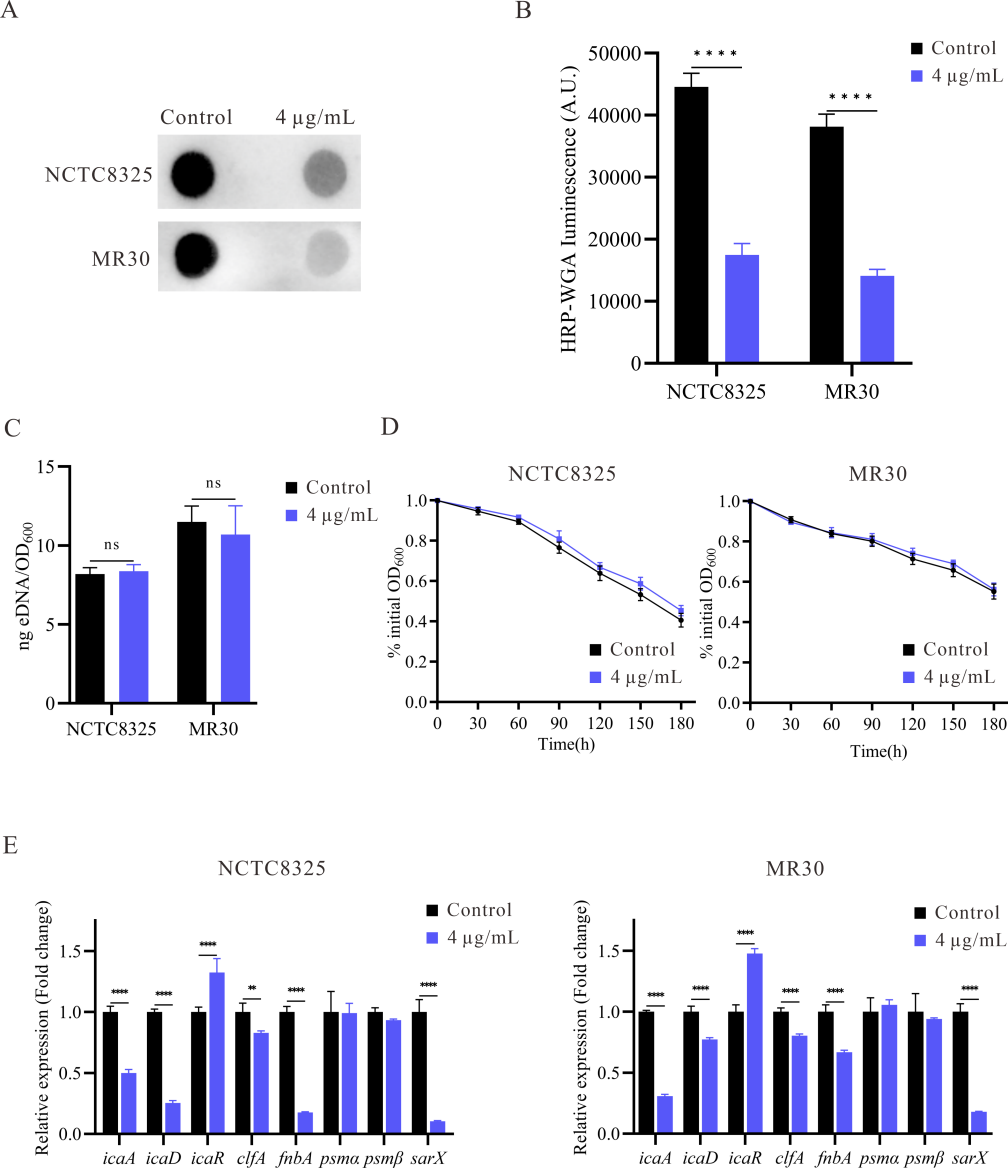
The effect of YH7 on the biofilm matrix and related gene expression of *S. aureus* strains. (**A**) Effect of YH7 on PIA production of *S. aureus*. PIA was semi-quantified using a dot blot assay with WGA (Wheat Germ Agglutinin). (**B**) Evaluation of gray values of PIA using Image J software. (**C**) Quantification of eDNA in *S. aureus* strains with or without YH7. (**D**) Effect of YH7 on autolysis ability of *S. aureus*. (**E**) RT-qPCR detection of the effect of YH7 on *S. aureus* biofilm-related genes expression. The relative expression level of the control was set as 1. Transcript levels were normalized to internal reference gene *gyrB*. Fold change values of the treated samples were calculated.

Extracellular PIA biosynthesis is pivotal for promoting intercellular adhesion and facilitating aggregation in *S. aureus* biofilms (37). To elucidate the impact of YH7 on the production of biofilm matrix, we utilized dot blot analysis to quantify the levels of extracellular PIA. Following treatment with 4 µg/mL YH7, there was a significant decrease in PIA content compared to the control group (*P* < 0.05) (Fig. 4A and B). Another factor that can promote biofilm formation is bacterial autolysis ability (38). eDNA is a crucial component of biological matrices. In staphylococcus biofilms, cell lysis serves as the primary mechanism for eDNA release. Therefore, we assessed the influence of YH7 on *S. aureus* eDNA release and self-digestion by inducing bacterial autolysis using Triton X-100. However, the eDNA levels in the YH7-treated and untreated strains were comparable, suggesting that YH7 had little influence on the autolysis ability of *S. aureus* (Fig. 4C). Furthermore, no significant alterations were observed in the autolysis ability of the isolates (NCTC8325 and MR30) when treated with 4 µg/mL of YH7 (Fig. 4D), suggesting that the inhibitory effect of YH7 on biofilm formation is not contingent upon the suppression of bacterial autolysis. Our combined data suggest that YH7 inhibits *S. aureus* biofilm formation by reducing PIA production but not by eDNA release.

### Inhibition of biofilm-related gene expression in *S. aureus* by YH7

To elucidate the underlying mechanism of YH7 in preventing *S. aureus* biofilm formation, we examined the transcription levels of various genes associated with biofilm formation using qRT-PCR analysis. The transcriptional levels of biofilm formation and adhesion genes (*icaA*, *icaD*, *clfA,* and *fnbA*), and the global regulatory gene *sarX* were significantly downregulated after 9 h of treatment (Fig. 4E). However, there were no significant changes in the expression of phenol-soluble modulins (*psmα* and *psmβ*).

### YH7 decreases *S. aureus* biofilm formation in a *sarX*-dependent fashion

**Fig 5:**
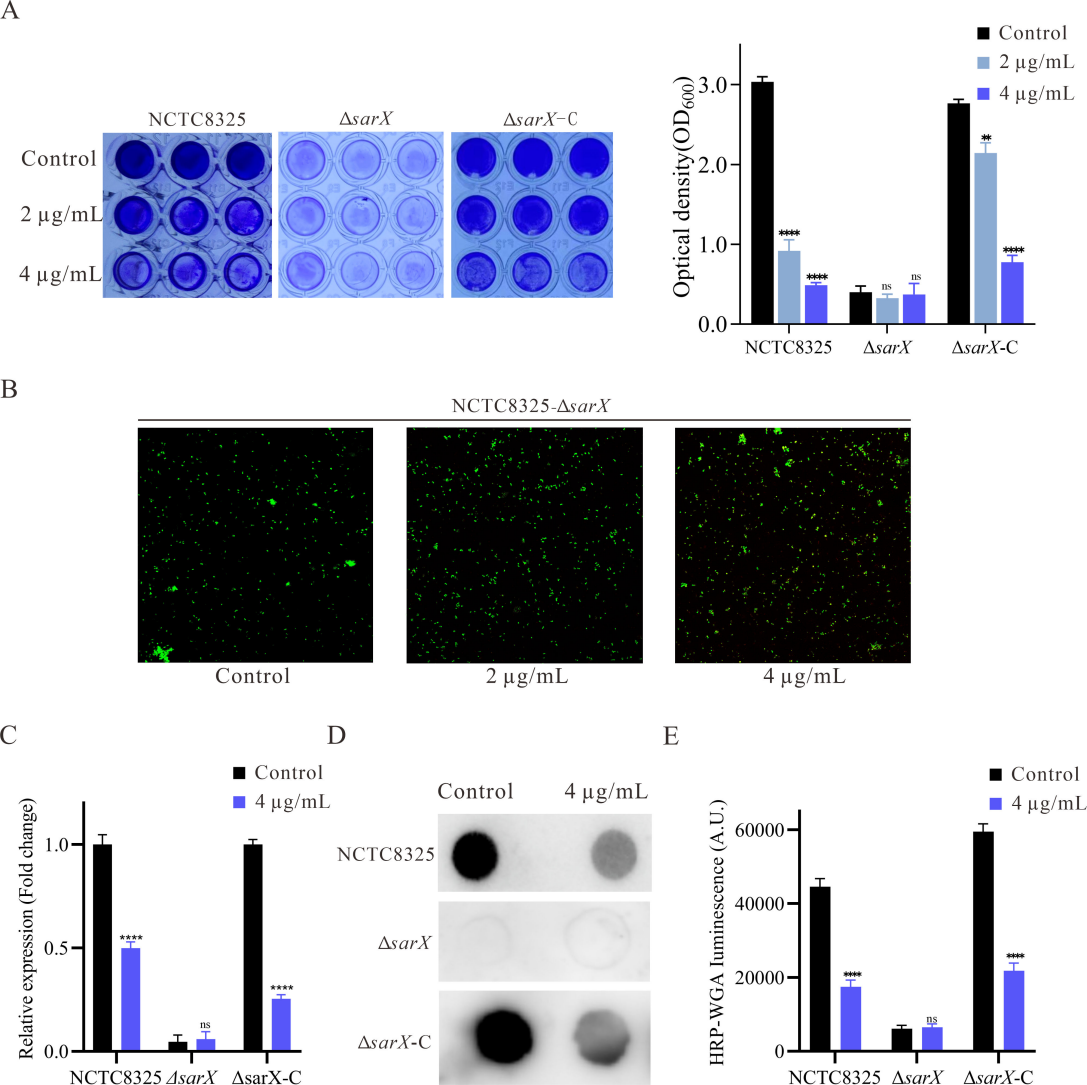
YH7 decrease *S. aureus* biofilm formation in a *sarX*-dependent fashion. (**A**) The biofilm formation of the NCTC8325 and Δ*sarX*, Δ*sarX*-C with or without YH7 (left), and the optical density values at 600 nm were measured (right). The biofilm formation image for NCTC8325 is the same as shown in Fig. 3A, as both were obtained from the same experiment. (**B**) The CLSM images of NCTC8325-Δ*sarX* in the presence of YH7 for 24 h. (**C**) The expression of *icaA* in NCTC8325 and Δ*sarX*, Δ*sarX*-C, with or without YH7. (**D**) The PIA production of the NCTC8325 and Δ*sarX*, Δ*sarX*-C with or without YH7. The PIA image for NCTC8325 is the same as shown in Fig. 4A, as both were obtained from the same experiment. (**E**) Evaluation of gray values of PIA using Image J software.

Previous research has suggested that *sarX* functions as an activator of the transcription of *icaADBC*, contributing to biofilm formation in *S. aureus* (39). Our previous studies have also suggested that *sarX* may promote biofilm formation by increasing the expression of the *ica* operon and production of PIA, rather than eDNA release (40). In this study, RT-qPCR results showed that *sarX*, *icaA,* and *icaD* were downregulated by YH7 treatment. Moreover, the antibiofilm effect of YH7 was attributed to its ability to reduce PIA production rather than eDNA release. This intriguing finding led us to hypothesize that YH7 exerts its influence through the modulation of *sarX*. To further investigate the influence of *sarX*-dependent YH7 on biofilm formation, we constructed NCTC8325 *sarX* knockout (Δ*sarX*) and complemented strains (Δ*sarX*-C) using allelic replacement (Fig. S1A). The success of these processes was confirmed by PCR and DNA sequencing (Fig. S1B and Fig. S1C). Notably, varying concentrations of YH7 had no impact on biofilm formation in the Δ*sarX* mutant (Fig. 5A). However, when the Δ*sarX* complementary mutant was treated with YH7, significant reduction in biofilm production was observed, similar to the WT strain. Considering the greatly reduced biofilm produced by the Δ*sarX* mutant and the poor sensitivity of crystal violet staining, we used CLSM to determine the impact of varying YH7 concentrations on Δ*sarX* biofilm formation. As shown in Fig. 5B, CLSM results indicate that varying YH7 concentrations appear to have no discernible effect on Δ*sarX* biofilm formation. Additionally, in the Δ*sarX* mutant, YH7 had no significant effect on *icaA* expression (Fig. 5C). We also observed that YH7 treatment did not attenuate PIA production in the Δ*sarX* mutant, despite overall lower PIA levels in this mutant (Fig. 5D and E). The *sarX* complementation mutant restored susceptibility to YH7-mediated attenuation of PIA production. These results demonstrate that YH7 inhibits *S. aureus* biofilm formation in a *sarX*-dependent manner.

## DISCUSSION

MRSA forms biofilms on medical devices, enabling it to develop resistance to antibiotics and evade the host immune system (8). Antibiotics that target the growth of planktonic cells can exert selective pressure on microorganisms, thereby inducing stress pathways and accelerating microbial evolution (41). The anti-virulence strategy, aimed at preventing biofilm formation without inhibiting bacterial growth, has attracted considerable interest (42). Small molecules are particularly promising because of their excellent stability, low toxicity, and minimal drug resistance. Notably, maleimide and diselenide scaffolds possess numerous desirable biological properties (15 – 17). In this study, a novel maleimide-diselenide hybrid (YH7) was successfully synthesized using a facile and metal-free reaction approach. YH7 exhibited remarkable antimicrobial activity against MSSA and MRSA in planktonic cultures and biofilms. Importantly, we evaluated the biocompatibility of YH7 *in vitro* and *in vivo*, as well as its potential to induce drug resistance in *S. aureus*. Hemolysis, cytotoxicity, and *G. mellonella* larval model assays demonstrated that YH7 at its antibacterial concentration (16 µg/mL) was non-toxic to rabbit red blood cells, A549 cells, BEAS-2B cells, and *G. mellonella* larvae. Drug resistance induction experiments revealed that YH7 has a lower propensity than rifampicin and ciprofloxacin to induce drug resistance in *S. aureus*.

High concentrations of YH7 (8 µg/mL) inhibited the growth of *S. aureus* in the logarithmic phase, whereas a sub-MIC of 4 µg/mL exhibited a negligible impact on the bacterial growth rate. To rule out the possibility that YH7 prevents *S. aureus* biofilm formation solely through its bactericidal efficacy, a sub-MIC of 4 µg/mL was chosen for the biofilm formation studies. The dynamic formation process of *S. aureus* biofilms involves three major stages: attachment, maturation, and dispersion (43). The initial attachment of *S. aureus* is the first step in successful infection of the host cell surface and subsequent biofilm formation (33). After initial attachment, bacterial cells produce ECM to facilitate intercellular binding. The mature stage is distinguished by the development of microcolonies that include the participation of various components such as PIA, eDNA, and proteins (44). PIA is a polymeric sugar molecule synthesized by the *ica* operon that acts as a glue-like substance that promotes bacterial adhesion and enables them to stick together (43). Inhibition of PIA/PNAG (poly-β(1-6)-N-acetylglucosamine) in an *ica*-dependent manner is a well-known mechanism for curtailing the formation of *S. aureus* biofilms (37). IcaR functions as a negative regulatory factor for the *ica* operon, and its inactivation results in the increased transcription of *icaAD* (45). Apart from PIA, eDNA is an essential component of *S. aureus* biofilm matrix and is released through autolysis (46). In this study, 4 µg/mL YH7 significantly inhibited *S. aureus* to solid surfaces. Consistently, YH7 considerably reduced MSSA and MRSA adhesion to the A549 cells. The anti-adhesive effect of YH7 has also been confirmed in mouse nasal colonization model. Moreover, our results show that the biofilm inhibitory effect of YH7 is achieved through the inhibition of PIA synthesis rather than by suppressing bacterial autolysis.

SarX, a key global regulator in *S. aureus*, promotes biofilm formation by transcriptionally activating the *ica* operon, which is necessary for PIA synthesis (39, 40). To explore the antibiofilm mechanism of YH7, RT-qPCR analysis was performed to assess the relevant gene expression levels associated with biofilm formation. The results showed that four genes (*icaA*, icaD, *clfA,* and *fnbA*) associated with bacterial biofilm formation and adhesion were significantly downregulated, except for *icaR*. Moreover, the global regulator *sarX* was downregulated upon YH7 treatment, which is consistent with the results of our phenotypic characterization. Biofilm dispersion is the terminal stage of the biofilm developmental cycle (47). Dispersion is mainly facilitated by the expression of PSMs (Phenol-soluble modulins), which allow bacteria to move to distant sites and form new biofilms (48). However, in isolates NCTC8325 and MR30, the expression levels of *psmα* and *psmβ* were not significantly altered, suggesting that YH7 had little or no effect on biofilm dispersion. Furthermore, despite that YH7 exhibited potent antibiofilm formation activity, it did not demonstrate the ability to eradicate established mature biofilms. These experimental findings demonstrated that YH7 effectively inhibited the formation of *S. aureus* biofilms by suppressing the expression of adhesion-related genes as well as the global regulatory gene *sarX*. YH7 effectively suppressed the initial bacterial adhesion and production of PIA, thus impeding biofilm formation.

In light of these findings and our qPCR results, we propose that the antibiofilm activity of YH7 may be dependent on the presence of *sarX*. To test this hypothesis, we generated a *sarX* knockout strain (NCTC8325-Δ*sarX*) and assessed the impact of YH7 on its biofilm formation capability. As expected, YH7 did not inhibit biofilm formation or PIA production in the Δ*sarX* strain, whereas it effectively suppressed biofilm formation and PIA production in the complemented strain. However, our research has certain limitations. Despite this study indicating that YH7 inhibits *S. aureus* biofilm formation in a *sarX*-dependent manner, the possibility of additional indirect or parallel pathways contributing to YH7’s inhibitory activity cannot be ruled out. Further validation of the specific molecular targets and pathways involved is still necessary.

In summary, the present study demonstrated that YH7 exhibits excellent antibiofilm activity against MSSA and MRSA. Preliminary mechanistic studies showed that YH7 exerts its antibiofilm effects in a *sarX-dependent* manner. Importantly, YH7 was confirmed to exhibit good *in vitro* and *in vivo* biosafety at antibacterial concentrations. Further *in vivo* infection models are required to evaluate its potential for clinical application in the management of *S. aureus* infections.

## SUPPLEMENTAL MATERIAL

Fig. S1 msphere.00564-23-s0001.pdf Identification of Δ*sarX* and Δ*sarX-C* strains.

ASM does not own the copyrights to Supplemental Material that may be linked to, or accessed through, an article. The authors have granted ASM a non-exclusive, world-wide license to publish the Supplemental Material files. Please contact the corresponding author directly for reuse.
