# Supplementary material for: Novel small-molecule compound YH7 inhibits the biofilm formation of Staphylococcus aureus in a sarX-dependent manner
Source: mSphere. 2024 Jan 3;9(1):e00564-23. doi: 10.1128/msphere.00564-23 (PMC10826350; doi:10.1128/msphere.00564-23)
Supplement: Fig. S1 — Identification of ΔsarX and ΔsarX-C strains. [file msphere.00564-23-s0001.pdf]

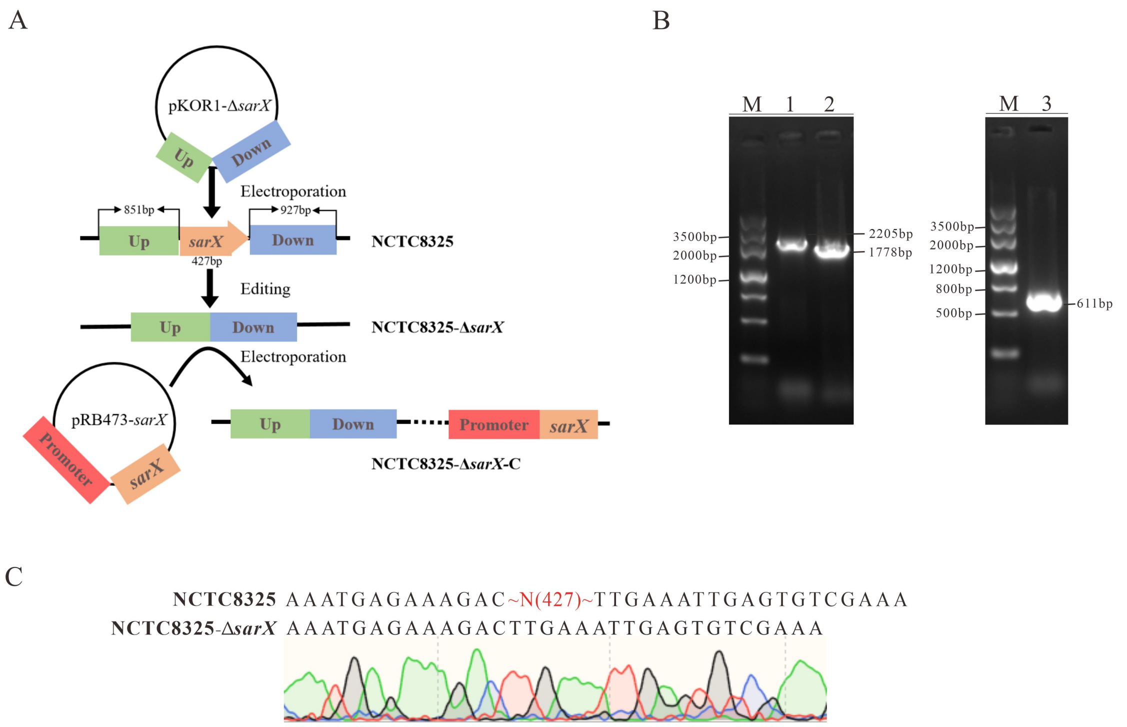

Figure S1. Identification of Δ*sarX* and Δ*sarX*-C strains.(A) Schematic model of construction of Δ*sarX* and Δ*sarX*-C. (B) PCR identification of Δ*sarX* and Δ*sarX*-C. M: DNA marker; 1: WT; 2: Δ*sarX*; 3: Δ*sarX*-C. (C) DNA sequencing result for the validation of Δ*sarX*.
